# Characterization of three *Francisella tularensis* genomes from Oklahoma, USA

**DOI:** 10.1099/acmi.0.000451

**Published:** 2023-06-14

**Authors:** Sai Narayanan, Brian Couger, Haley Bates, Sushim Kumar Gupta, Jerry Malayer, Akhilesh Ramachandran

**Affiliations:** ^1^​ Oklahoma Animal Disease Diagnostic Laboratory, College of Veterinary Medicine, Oklahoma State University, 1950 W Farm Rd, Stillwater, OK 74078, USA; ^2^​ Brigham and Women’s Hospital, Harvard Medical School, 75 Francis St., Boston, MA 02115, USA; ^3^​ Yale School of Nursing, 400 W Campus Dr., Orange, CT 06477, USA; ^4^​ College of Veterinary Medicine, Oklahoma State University, 208 S McFarland St., Stillwater, OK 74078, USA

**Keywords:** *F. tularensis*, tularaemia, pathogen characterization, Oklahoma (USA), genome characterization

## Abstract

*
Francisella tularensis *, the causative agent for tularaemia, is a Tier 1 select agent, and a pan-species pathogen of global significance due to its zoonotic potential. Consistent genome characterization of the pathogen is essential to identify novel genes, virulence factors, antimicrobial resistance genes, for studying phylogenetics and other features of interest. This study was conducted to understand the genetic variations among genomes of *F. tularensis* isolated from two felines and one human source. Pan-genome analysis revealed that 97.7 % of genes were part of the core genome. All three *F. tularensis* isolates were assigned to sequence type A based on single nucleotide polymorphisms (SNPs) in *sdhA*. Most of the virulence genes were part of the core genome. An antibiotic resistance gene coding for class A beta-lactamase was detected in all three isolates. Phylogenetic analysis showed that these isolates clustered with other isolates reported from Central and South-Central USA. Assessment of large sets of the *F. tularensis* genome sequences is essential in understanding pathogen dynamics, geographical distribution and potential zoonotic implications.

## Data Summary

**Table:** 

Strain	Assembly accession no.	BioSample ID	BioProject
OADDL_FT1-Narayanan	GCA_022500845.1	SAMN24591981	PRJNA793874
OADDL_FT2-Couger	GCA_022500885.1	SAMN24592384	PRJNA793874
OADDL_FT3-Bates	GCA_022500855.1	SAMN24592125	PRJNA793874

**Impact Statement**

*
Francisella tularensis* is the causative organism of tularaemia, a deadly disease in over 100 species. Genome analysis and frequent genome surveillance would be helpful to assess the genomic alterations and their effect on pathogenicity, virulence and trends in antimicrobial resistance. This study aims to assess three genomes of *F. tularensis *, giving insight into the dynamics of this pathogen. Whole genomes of bacteria isolated from one human (archived), and two felines were sequenced and analyzed in this study.

## Introduction

*Francisella tularensis* is a Gram-negative coccobacillary intracellular bacterium belonging to the class *Gammaproteobacteria. F. tularensis* is the causative agent of tularaemia, a vector-borne zoonotic disease commonly called ‘rabbit fever’. Tularaemia has been reported in all states of the USA except Hawaii [1]. Due to its high infectivity and potential to be aerosolized, *F. tularensis* is considered a bioterrorism agent and has been classified as a Tier 1 select agent [1–3] by the Centers for Disease Control and Prevention (CDC, USA).

*F. tularensis* has a broad host range, and has been reported from more than 300 animal species [4–7] including mammals, birds, amphibians and invertebrates [6, 7] many of which can serve as a major pathway for zoonosis. Various vectors such as ticks [4, 8] and flies [9] aid in pathogen transmission in the USA. Major tick vectors include *Amblyomma* sp., *Dermacentor* spp. [10], *Haemaphysalis* spp. and *Ixodes* spp. [11, 12]. Flies belonging to the family Tabanidae have also been identified as vectors for *F. tularensis* [2, 3, 9]. Besides arthropod vectors, a few birds, mammals and inanimate substances can also act as sources for pathogen transmission to humans. Rabbits, hares, rats, voles and prairie dogs have been reported to serve as reservoir hosts and transmitters of *F. tularensis* [2, 3, 9, 13–15]. Felines, lagomorphs, and rodents have been identified to be important sources of infection for humans [9, 15]. Rabbits, by means of predation can serve as a source to infect cats [13, 15, 16]. Birds, especially Galliformes such as the grouse, have also been implicated as carriers of ticks that can transmit Tularaemia to humans [7]. Besides rare clinical reports [17, 18], tularaemia is not commonly reported in birds. In one case, *F. tularensis* was also reported to be transmitted by organ transplantation [19].

*F. tularensis* is categorized into different subspecies: *F. tularensis* subsp. *tularensis* (Type A), *F. tularensis* subsp. *holarctica* (Type B) and *F. tularensis* subsp. *mediaasiatica* [20]. Type A *F. tularensis* is sub-typed into Type A1 and Type A2. Type A1 is further sub-classified as Type A1a and Type A1b [15, 21]. Of these subtypes, Type A1b has been reported to be significantly more virulent in humans than the others [21]. Both Type A and Type B infections are reported in North America while Type B infections are reported more commonly in Asia and Europe [22]. In the USA, the highest incidence of tularaemia has been reported from South Dakota, Nebraska, Kansas, Missouri, Arkansas and Oklahoma [8, 9, 23].

Genome characterization of bacteria is essential to understand their pathogenic features and zoonotic implications. Assessment of alterations in pathogen genomes can also help in better understanding their provenance and geographical distribution for source attribution studies. In this study, we assessed genome sequences of *F. tularensis* subsp *. tularensis* isolated from one human and two felines in Oklahoma, USA. The sequences were compared to assess: variations among the isolates, presence of virulence factors, presence of antimicrobial resistance genes, and their phylogenetic relationship to a wide range of previously reported isolates of *F. tularensis *.

## Methods

### Bacterial identification and isolation

Spleen samples from two felines (*Felis domesticus*) with suspect postmortem lesions for tularaemia were received for diagnostic confirmation by PCR of *F. tularensis* ISFtu2 gene [24]. Following PCR confirmation, under Biosafety level 3 (BSL-3) conditions, spleen samples from two felines and one archived human isolate were plated on chocolate agar media (Hardy Diagnostics) and incubated at 37 °C for 48 h to isolate and grow *F. tularensis.*

### Sequencing, genome assembly and annotation

Bacterial DNA was extracted using an E.Z.N.A. bacterial DNA extraction kit (Omega Bio-Tek). The quality and quantity of the isolated genomic DNA (gDNA) were determined using a NanoDrop One spectrophotometer (Thermo Fisher Scientific) and with a Qubit 4 fluorometer (Thermo Fisher Scientific) using the Qubit dsDNA BR kit (Invitrogen). The purified gDNA was sent to the Yale Center for Genome Analysis for PacBio sequencing. PacBio reads were assembled using a high-noise single-molecule sequencing assembler, Flye [25]. Assembled genomes from PacBio sequencing data were used for downstream analysis. Prokka [26] was used for whole genome annotation. Average nucleotide identity (ANI) was calculated using a web-based ANI calculator (https://www.ezbiocloud.net/tools/ani). Closely related genes that share phylogeny with hypothetical genes were identified using blastp.

### Pangenome and phylogenetic analysis

Pangenome analysis was performed using Roary [27]. Roary has an in-built alignment pipeline based on –mafft [28], which was used for core genome alignment. The .gff files of the three *F. tularensis* generated from prokka annotation were used to identify core and accessory genes in the studied *F. tularensis* genomes. Additionally, 243 genome sequences for *F. tularensis* (which included 146 *F *. *tularensis* subsp. *holarctica *, four *F *. *tularensis* subsp. *mediasiatica *, 14 *F *. *tularensis* subsp. *novicida *, 32 *F *. *tularensis* subsp. *tularensis* and 47 *F *. *tularensis* with no data on subspecies) (Table S1, available in the online version of this article) were downloaded from NCBI GenBank database and annotated using prokka [26]. Core genome alignment was performed using Roary with –mafft option for all the 243 genomes from the NCBI and three *F. tularensis* genomes sequenced in this study. The core genome aligned file was used to reconstruct an approximate maximum likelihood phylogenetic tree using FastTree [29], with a Generalized Time Reversible (GTR) model, and a bootstrap value of 100. Based on the phylogenetic analysis, respective closest neighbours for the three genomes in this study were identified and their geographical locations were recorded and these genomes were analysed for similarity. OrthoVenn2 [30] (https://orthovenn2.bioinfotoolkits.net/) was used to assess the similarity between genomes, identify clusters of orthologous genes and singleton genes between the three draft genomes as well as between the draft genomes and genomes with the highest phylogenetic similarity.

The coding sequence for the gene succinate dehydrogenase (*sdhA*) was identified. The gene *sdhA* has been used for typing *F. tularensis* based on nucleotide changes at position 465. The presence of G or A at position 465 in *sdhA* is characteristic for type B and type A respectively [31, 32].

### Genetic elements

Comprehensive Antibiotic Resistance Database (RGI-CARD) [33], ResFinder [34] and ARG-ANNOT [35] were used to identify antimicrobial resistance genes. Mutations in the *gyrA* sequences were analysed using blastn [36]. VF Analyzer available with the Virulence Factor Database (VFDB) [37] was used to predict ORFs (Open Reading Frames) for different virulence factors. ISFinder [38] was used to identify insertion sequences. CanSNPer was used to identify and classify the subtypes of *F. tularensis* [39, 40].

## Results

### Genome assembly and gene annotation

Two feline isolates (OADDL_FT1-Narayanan (FT1) and OADDL_FT2-Couger (FT2)] and a human isolate [OADDL_FT3-Bates (FT3)] were sequenced and assembled. In brief, all three isolates (OADDL-FT) were assembled into two contigs with a genome size ranging between ~1.89 and 1.96 Mb with an average GC content of 32.23 %. The ANI between these microbes was 99.97%, indicating that these genomes are highly similar. However, the genome coverage of these microbes for ANI was <80 %, which indicated the diversity in OAADL-FT isolates. The genome sequences have been deposited in the NCBI GenBank database and their assembly accession numbers along with genome statistics are listed in Table 1. A total of 1997, 1953 and 2007 genes that includes 61, 52 and 62 RNA genes in FT1, FT2 and FT3 respectively, were identified. Roary [27] output of OAADL-FT was assessed for core and accessory genes.

**Table 1.** Total genome length, assembly metrics and final number of contigs along with number of Coding Sequences (CDS) predicted from all the three draft genomes sequenced in this study

Draft genome	Assembly accession no.	Total genome length	N50	No. contigs	Coverage	No. of CDS predicted
FT1	GCA_022500845.1	1 958 085 bp	1 531 263	2	498×	1997
FT2	GCA_022500885.1	1 893 091 bp	1 499 812	2	617×	1953
FT3	GCA_022500855.1	1 960 048 bp	1 532 941	2	523×	2007

### Phylogenetic analysis

**Fig. 1.**
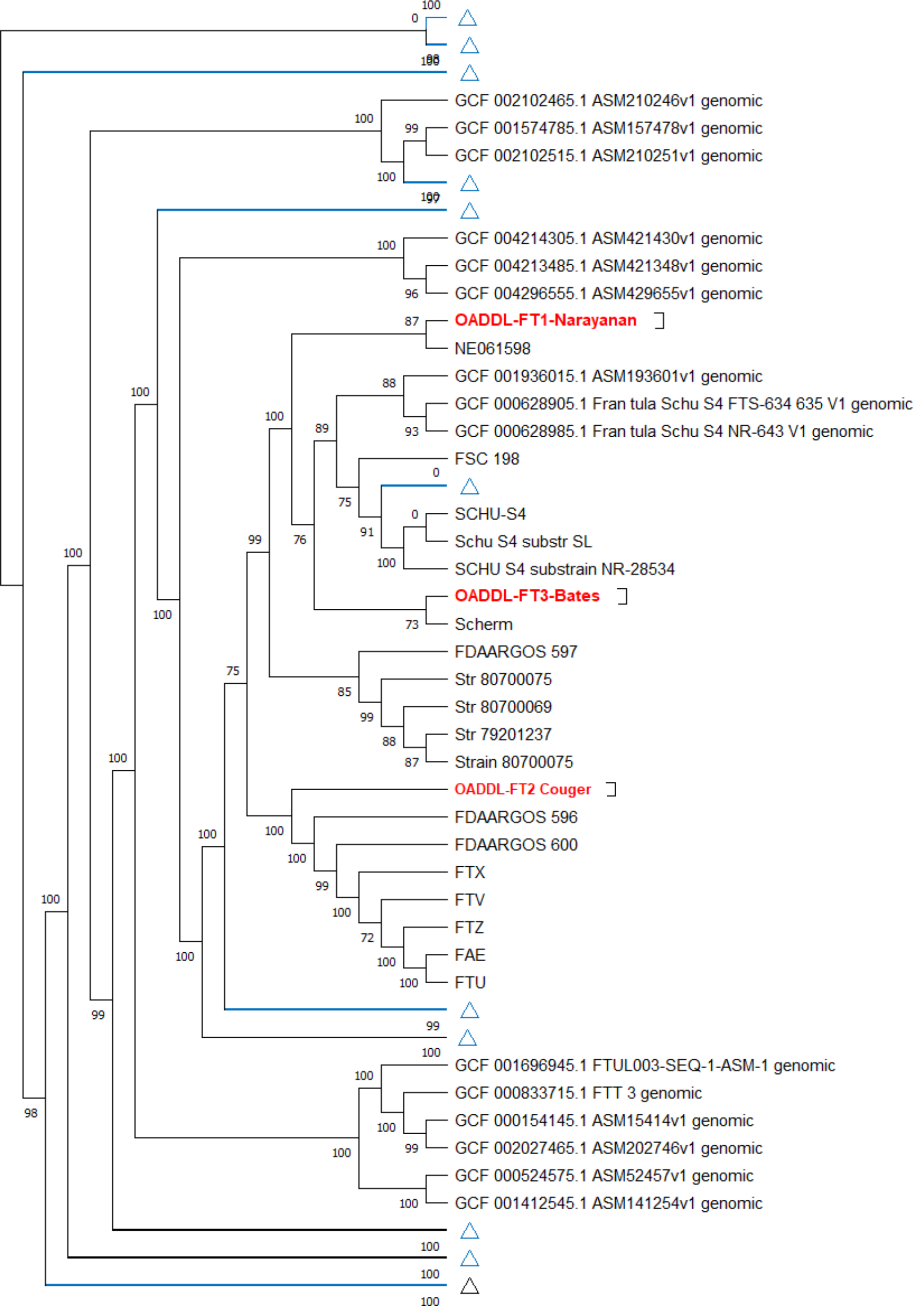
Phylogenetic analysis (rooted) of the three draft genomes and 243 genomes from NCBI. Organisms that are not in the same clade with the draft genomes have been minimized for visualization. A bootstrap value of 100 was used to generate the phylogenetic tree.

Phylogenetic analysis with core genes indicated that the OADDL-FT strains clustered into three distinct clades with *F. tularensis* genomes from GenBank (Fig. 1). FT1 clustered with the isolate reported from Nebraska, USA (strain: NE061598) (Fig. 1). FT2 shared a close common clade with isolates reported from multiple rabbits in Illinois, USA (strains: FDAARGOS_596, FTX, FTV, FTZ, FAE, FTU) (Fig. 1) and an isolate from Ireland (strain: FDAARGOS_600). OADDL_FT3-Bates, the human isolate, shared a close common clade with the human isolate reported from Ohio, USA (strain: Scherm) (Fig. 1).

**Fig. 2.**
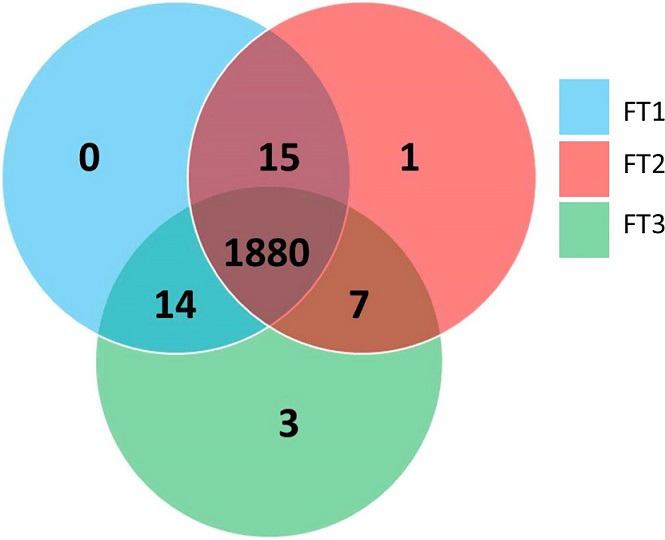
Assessment of clusters of orthologous groups between OADDL-FT genomes. FT1=OADDL_FT1-Narayanan; FT2=OADDL_FT2-Couger; FT3=OADDL_FT3-Bates.

A total of 1880 shared clusters of orthologous genes (core genes) were identified between the OADDL-FT genomes (Fig. 2). Fifteen shared clusters were identified between FT1 and FT2. Fourteen and seven shared clusters were identified between FT1 and FT3 and FT2 and FT3 (Fig. 2) respectively. Only a single gene in FT2 and three genes in FT3 were not shared with the genes of the other two OADDL-FT genomes (Fig. 2). The genes in these non-shared clusters were annotated for transposases (FT2), pathogen determinant pdpC protein domain (FT3) and hypothetical proteins (FT3). From a total of 1920 clusters identified across the three draft genomes, 72 clusters were derived from orthologous genes across samples and 1848 clusters were derived from single-copy genes.

### Genetic elements

VFDB was used to predict and identify ORFs for possible virulence factors in the draft genomes, using *F. tularensis* subsp. *tularensis* SCHU S4 as a representative genome for comparison. These virulence factors are listed in Table S2. Various virulence factors belonging to different classes, namely Adherence and Invasion factors (EF-Tu, FsaP, Type IV pili), Intracellular survival factors (acid phosphatases, DipA, OmpA, RipA), Iron uptake factors (ferrous iron-transport system, *Francisella* siderophore locus), Magnesium uptake factors (FmvB), Nutritional virulence factors (arginine transporter, asparagine transporter, biotin metabolism, cysteine acquisition, glutamate transporter, isoleucine transporter, purine and pyrimidine biosynthesis), Secretion systems (T6SS), Serum resistance and immune evasion factors (capsular factors and LPS) and Toxins were annotated. All OADDL-FT genomes shared all but one virulence factor with *F. tularensis* subsp. *tularensis* SCHU S4. The three OADDL-FT genomes had an annotated toxin virulence factor, phytotoxin phaseolotoxin (*cysC1*), which was absent in SCHU S4. Phaseolotoxin is a plant toxin commonly produced by *Pseudomonas* spp. and is known to induce chlorosis in plants [41]. Various bacterial factors for host invasion during phagosomal escape [42] such *as iglC, iglD, pdpA, iglI, vgrG, carA, carB, pyrB, acpA, acpB, acpC* and *hapA* and bacterial factors for cytosolic replication [42] such as *dipA, iglD, purMCD, ggt, ripA, iglA, iglB* and *pdpB* were also identified (Table S2).

Using databases from ISfinder (https://isfinder.biotoul.fr/), insertion sequences were annotated. The insertion sequences that had blast alignments to segments of the draft genomes are listed in Table 2.

**Table 2.** Subtyping of the draft genomes, Insertion sequences and the number of Tandem Repeats sequences annotated in each draft genome

Draft genomes	Assembly accession no.	CanSNPer annotation	Insertion sequences annotated using ISfinder
FT1	GCA_022500845.1	A.1.14	ISFtu1, ISFtu2, ISFtu2, ISFtu3, ISFtu5, ISFtu6, ISFtu7, ISFw10
			ISFtu1, ISFtu4, ISFtu2, ISFtu3, ISFtu7, ISFtu6, ISFop2
FT2	GCA_022500885.1	A.1.11	ISFtu5, ISFtu1, ISFtu2, ISFtu6, ISFop2, ISFtu3, ISFw10, ISFtu7.
			ISFtu1, ISFtu4, ISFtu2, ISFtu3, ISFtu7, ISFtu6, ISFop2
FT3	GCA_022500855.1	A.1.13	ISFtu1, ISFtu4, ISFtu2, ISFtu3, ISFtu7, ISFtu6
			ISFtu5, ISFtu1, ISFtu2, ISFtu6, ISFop2, ISFtu3, ISFw10, ISFtu7

The coding sequence for the gene succinate dehydrogenase (*sdhA*) was identified and assessed for typing. Our analysis confirmed the presence of A at nucleotide position 465 of the *sdhA* gene in all three genomes, which confirmed that OADDL-FT isolates are Type A *F. tularensis,* which was further validated by CanSNPer. The CanSNPer pipeline aims to identify single nucleotide polymorphisms (SNPs) of diagnostic significance. Based on CanSNPer typing, the genomes FT1, FT2 and FT3 were assigned as types A.1.14, A1.11, and A.1.13 respectively. Two antibiotic resistance genes, *bla*FTU-1 and *bla*A, were detected in all OADDL-FT genomes. blaFTU-1 codes for a class A beta lactamase and confers resistance to beta-lactams in *F. tularensis* [43]. We further explored the OADDL-FT *gyrA* gene for chromosomal mutations that confer resistance to fluoroquinolone [44]. No known mutations in *gyrA* were observed in OADDL-FT genomes [45]. However, when compared to the reference *F. tularensis gyrA* gene (NC_007880), we observed multiple mutations in the OADDL-FT *gyrA* gene that lead to amino acid substitutions at positions 395 (Glu-395-Lys), 486 (Ile-486-Val), 501 (Cys-501-Arg) and 670 (Thr-670-Lys).

The genomes have been submitted to NCBI GenBank under BioProject ID PRJNA793874, BioSample IDs SAMN24591981, SAMN24592384 and SAMN24592125, and assembly accession numbers GCA_022500845.1, GCA_022500885.1 and GCA_022500855.1 for FT1, FT2 and FT3 respectively.

## Discussion

*F. tularensis* is a category-A bioterrorism agent. Tularemia, the disease condition caused by *F. tularensis,* is usually fatal if not treated immediately. Tularaemia can be transmitted to humans by means of bites from ticks or deer flies, contact with infected animals, drinking contaminated water, and inhalation of contaminated aerosols [9, 23]. The advent of modern technology such as whole genome sequencing has made characterizing pathogens easier and in understanding the association between genetic information and characteristics such as pathogen distribution, dynamics and transmission patterns. The south-central states in the continental USA, namely Arkansas, Louisiana, Missouri, Oklahoma and South Dakota, have reported the highest incidences of *F. tularensis* from 1990 to 2000 [46]. Contact with wild Lagomorpha and Rodentia is an established route of transmission for Tularemia in cats [13, 16]. Similar history was reported in both the felines in this study.

Extensive studies in the past have determined the pathogenesis of *Francisella* spp. inside mammalian hosts. The bacteria enter and reside in macrophages otherwise classified as the *Francisella* Containing Phagosome. There they undergo extensive replication, resulting in cell swelling, cell death and release of bacteria, which may further attack additional macrophages [42]. Ultimately, endotoxaemia and bacteraemia due to massive lysis of cells results in the death of the mammalian host.

This study reports genomic findings of three archived *F. tularensis* isolates that were sequenced. Genomic sequences of two isolates from felines and one from a human were analysed. A total of 1880 genes out of 1920 genes were observed to be core genes in the OADDL-FT genomes. The presence of a large number of similar genes between the three bacterial genomes was expected given the close genomic relationship between the three strains. This was further supported by CanSNPer annotations. The OADDL-FT genomes sequenced in this study were of Type A.1.

The maximum likelihood phylogenetic tree with a GTR model of the OADDL-FT genomes and 243 other *F. tularensis* genomes indicated that these OADDL-FT genomes grouped more commonly with the isolates reported from Central USA (Fig. 1). The lack of genetic diversity across geographical areas makes it hard to characterize this pathogen as endemic to a state or political border inside the continental USA. The feline isolates from this study shared close common ancestors with isolates reported from Illinois and Nebraska. The human isolate shared a close common ancestor with an isolate reported from Ohio, USA. *Amblyomma americanum* [16, 47], a known tick vector for *F. tularensis,* is known to be widely distributed in these regions. One of the previously reported *Francisella* isolates from Oklahoma (strain: FDAARGOS_596) shared a close common ancestor with high similarity to FT2, one of the feline isolates.

It has been established that many *Francisella* species contain gene clusters that are homologous to a set of gene clusters of *Neisseria* and *Pseudomonas* [48–50]. These also include Type IV pili, which are essential for bacterial adhesion, aggregation, twitching motility, and DNA uptake [51]. One of the toxins identified by the VF analyser that was absent in SCHU S4 was *cysC1* – Phytotoxin phaseolotoxin. Based on previous studies, phaseolotoxin is a toxic agent for plants [52] secreted by *Pseudomonas* spp., and there are no known reports of phaseolotoxin pathogenicity in animals. Further experimentation will be needed to confirm the presence and function of the gene in OADDL-FT genomes. The three draft genomes shared many virulence factors with *F. tularensis* subsp. *tularensis* SCHU S4, indicating similar pathogenicity between these isolates. The annotation and identification of bacterial factors needed for phagosomal escape and cytosolic replication further emphasize the pathogenic potential of these isolates sequenced in this study.

Frost *et al*. [53] discussed that: ‘Mobile genetic elements (MGEs) are segments of DNA that encode enzymes and other proteins that mediate the movement of DNA within genomes (intracellular mobility) or between bacterial cells (intercellular mobility). Intercellular movement of DNA takes three forms in prokaryotes: transformation, conjugation and transduction.’ In general, MGEs include insertion sequences, phage sequences, conjugative transposons, integrons and unit transposons [53]. Insertion sequences are known to carry ORFs that encode transposases [54]. These insertion sequences usually result in interruption of a gene or alter genetic regulation, resulting in expression of a gene or inactivation [55]. MGEs aid in transmission of pathogenic components such as virulence factors, AMR genes etc. [53]. ISFtu1, ISFtu2, ISFtu3, ISFtu4, ISFtu5, ISFtu6, ISFtu7, ISFtu10, ISFop2 and ISFw10 were identified in all draft genomes in this study.

Genome analysis for antibiotic resistance genes revealed the presence of two β-lactamase (*bla*FTU-1 and *bla*A) genes in all the OADDL-FT isolates. These genes are intrinsic in the genus *Francisella* and *bla*FTU-1 confers narrow-spectrum resistance to β-lactams in most *Francisella* strains [43, 56]. Amino acid substitutions in DNA gyrase are the main mechanisms of fluoroquinolone resistance in a broad spectrum of bacterial genera including *Francisella* [45, 57]. Further experimentation will be needed to confirm if the aminoacid substitiuitions detected in *gyrA* genes of all OADDL-FT genomes, are associated with fluoroquinolones resistance.

The annotation of genetic elements such as insertion sequences, AMR genes and virulence factors underscore the need for regular sequencing of *Francisella* spp. to better understand bacterial evolution and dynamics.

## Supplementary Data

Supplementary material 1
