# Supplementary material for: Characterization of three Francisella tularensis genomes from Oklahoma, USA
Source: Access Microbiol. 2023 Jun 14;5(6):acmi000451. doi: 10.1099/acmi.0.000451 (PMC10323801; doi:10.1099/acmi.0.000451)
Supplement: Supplementary material 1 [file acmi-5-451-s001.pdf]

Supplementary Table 1: List of genomes used for phylogenetic comparison with draft genomes. (Source: NCBI, Date Accessed: 01.04.2022).

#Organism Name	HOST (UNK =unknown)	Location	Strain	BioSample	BioProject	Assembly
Francisella tularensis	Human	OR	FDAARGOS_595	SAMN10228575	PRJNA231221	GCA_003955815.1
Francisella tularensis	Human	IN	FDAARGOS_598	SAMN10228578	PRJNA231221	GCA_003955795.1
Francisella tularensis	Human	UT	FDAARGOS_599	SAMN10228579	PRJNA231221	GCA_003955775.1
Francisella tularensis	Hare	Germany	12T0050_FLI	SAMN08201031	PRJNA422969	GCA_002886065.1
Francisella tularensis	Human	USA	Schu4 F. tul Mut-127	SAMN04378279	PRJNA307317	GCA_001936015.1
Francisella tularensis	Human	China	T01	SAMN03799293	PRJNA288396	GCA_001412545.1
Francisella tularensis	UNK	Ireland	FDAARGOS_600	SAMN10228580	PRJNA231221	GCA_003797955.1
Francisella tularensis	UNK	Ireland	FDAARGOS_247	SAMN04875573	PRJNA231221	GCA_002082235.2
Francisella tularensis	UNK	KS	FDAARGOS_597	SAMN10228577	PRJNA231221	GCA_003798025.1
Francisella tularensis	UNK	OK	FDAARGOS_596	SAMN10228576	PRJNA231221	GCA_003798065.1
Francisella tularensis	UNK	USA	Larsen	SAMN02870009	PRJNA244555	GCA_000742095.1
Francisella tularensis	Rabbit	IL	FTZ	SAMN02887095	PRJNA244566	GCA_000741935.1
Francisella tularensis	Rabbit	IL	FAE	SAMN02869888	PRJNA244554	GCA_000742075.1
Francisella tularensis	Rabbit	IL	FTX	SAMN02870078	PRJNA244562	GCA_000742145.1
Francisella tularensis	Rabbit	IL	FTU	SAMN02870034	PRJNA244560	GCA_000742155.1
Francisella tularensis	UNK	Russia	15NIIEG	SAMN04606331	PRJNA317365	GCA_001611815.3
Francisella tularensis	Rabbit	IL	FAC	SAMN02867633	PRJNA244553	GCA_000746455.1
Francisella tularensis	Rabbit	IL	FTV	SAMN02870045	PRJNA244561	GCA_000742015.1
Francisella tularensis	Human	Norway	NO-12/2011	SAMN02870486	PRJNA253522	GCA_001574785.1
Francisella tularensis	Human	Norway	NO-11/2011	SAMN02870485	PRJNA253522	GCA_001574585.1
Francisella tularensis	Human	Norway	NO-6/2011	SAMN02870480	PRJNA253522	GCA_001574705.1
Francisella tularensis	Human	Norway	NO-17/2011	SAMN02870491	PRJNA253522	GCA_001574655.1
Francisella tularensis	Human	Norway	NO-14/2011	SAMN02870488	PRJNA253522	GCA_001574605.1
Francisella tularensis	Human	Norway	NO-13/2011	SAMN02870487	PRJNA253522	GCA_001574795.1
Francisella tularensis	Human	Norway	NO-4/2011	SAMN02870478	PRJNA253522	GCA_001574495.1
Francisella tularensis	Human	Norway	NO-2/2011	SAMN02870476	PRJNA253522	GCA_001574775.1
Francisella tularensis	Human	Norway	NO-9/2011	SAMN02870483	PRJNA253522	GCA_001574565.1
Francisella tularensis	Human	Norway	NO-10/2011	SAMN02870484	PRJNA253522	GCA_001574755.1
Francisella tularensis	Human	Norway	NO-5/2011	SAMN02870479	PRJNA253522	GCA_001574695.1
Francisella tularensis	Human	Norway	NO-7/2011	SAMN02870481	PRJNA253522	GCA_001574735.1
Francisella tularensis	Human	Norway	NO-3/2011	SAMN02870477	PRJNA253522	GCA_001574475.1
Francisella tularensis	Human	Norway	NO-8/2011	SAMN02870482	PRJNA253522	GCA_001574555.1

Francisella tularensis	Human	Norway	NO-15/2011	SAMN02870489	PRJNA253522	GCA_001574635.1
Francisella tularensis	Human	Norway	NO-1/2011	SAMN02870475	PRJNA253522	GCA_001574485.1
Francisella tularensis	Human	Norway	NO-16/2011	SAMN02870490	PRJNA253522	GCA_001574835.1
Francisella tularensis	Human	Turkey	FDC203	SAMN02886898	PRJNA253675	GCA_001574885.1
Francisella tularensis	Human	Norway	NO-18/2011	SAMN02870492	PRJNA253522	GCA_001574675.1
Francisella tularensis	UNK	Turkey	FDC202	SAMN02886897	PRJNA253675	GCA_001574945.1
Francisella tularensis	UNK	Turkey	FDC200	SAMN02886895	PRJNA253675	GCA_001574855.1
Francisella tularensis	UNK	Turkey	FDC204	SAMN02886899	PRJNA253675	GCA_001574905.1
Francisella tularensis	UNK	Japan	O'HARA	SAMN04260149	PRJNA257008	GCA_001696945.1
Francisella tularensis	UNK	Turkey	FDC201	SAMN02886896	PRJNA253675	GCA_001574865.1
Francisella tularensis	UNK	Turkey	FDC205	SAMN02886900	PRJNA253675	GCA_001574935.1
Francisella tularensis	Tick	Russia	503	SAMN04260148	PRJNA257008	GCA_001696925.1
Francisella tularensis	Human	Russia	RUSS	SAMN04260147	PRJNA257008	GCA_001696935.1
Francisella tularensis	Human	Russia	MAX	SAMN04260150	PRJNA257008	GCA_001696955.1
Francisella tularensis 99A-2628	Human	USA	99A-2628	SAMN02261485	PRJNA212941	GCA_000478845.1
Francisella tularensis subsp. holarctica	Water	MT	425	SAMN03251848	PRJNA240117	GCA_000833515.1
Francisella tularensis subsp. holarctica	Musk Rat	VT	VT68	SAMN03251846	PRJNA240115	GCA_000833495.1
Francisella tularensis subsp. holarctica	UNK	USA	FTT_1	SAMN03023737	PRJNA242267	GCA_000833235.1
Francisella tularensis subsp. holarctica	Primate	USA	OR96-0246	SAMN03486587	PRJNA281242	GCA_001044315.2
Francisella tularensis subsp. holarctica	Wine	Germany	Fth-Must	SAMN08011029	PRJNA417909	GCA_003612815.1
Francisella tularensis subsp. holarctica	UNK	OH	JAP	SAMN03252734	PRJNA240114	GCA_000833715.1
Francisella tularensis subsp. holarctica	Tick	Switzerland	FT16C-B1	SAMN03774942	PRJNA286987	GCA_002027785.1
Francisella tularensis subsp. holarctica	Human	Sweden	FSC247	SAMN03773988	PRJNA285145	GCA_002100255.1
Francisella tularensis subsp. holarctica	Human	Japan	FSC599	SAMN03774150	PRJNA285145	GCA_002100685.1
Francisella tularensis subsp. holarctica	UNK	France	FSC027	SAMN03773913	PRJNA285145	GCA_002100705.1
Francisella tularensis subsp. holarctica	UNK	France	FSC029	SAMN03773915	PRJNA285145	GCA_002100265.1
Francisella tularensis subsp. holarctica	European Hare	Italy	FSC031	SAMN03773917	PRJNA285145	GCA_002100585.1
Francisella tularensis subsp. holarctica	Hare	Switzerland	B.91	SAMN08108686	PRJNA286987	GCA_002857505.1

Francisella tularensis subsp. holarctica	Hare	Switzerland	B.85	SAMN08108685	PRJNA286987	GCA_002857515.1
Francisella tularensis subsp. holarctica	Mouse	Switzerland	B.87	SAMN08108681	PRJNA286987	GCA_002857605.1
Francisella tularensis subsp. holarctica	Hare	Switzerland	B.85	SAMN08108679	PRJNA286987	GCA_002857635.1
Francisella tularensis subsp. holarctica	Hare	Switzerland	B.61	SAMN08108678	PRJNA286987	GCA_002857665.1
Francisella tularensis subsp. holarctica	Hare	Switzerland	B.90	SAMN08108677	PRJNA286987	GCA_002857705.1
Francisella tularensis subsp. holarctica	UNK	Austria	FSC555	SAMN03774138	PRJNA285145	GCA_002100665.1
Francisella tularensis subsp. holarctica	UNK	Spain	FSC456	SAMN03774111	PRJNA285145	GCA_002100275.1
Francisella tularensis subsp. holarctica	UNK	France	FSC028	SAMN03773914	PRJNA285145	GCA_002100245.1
Francisella tularensis subsp. holarctica	UNK	France	FSC026	SAMN03773912	PRJNA285145	GCA_002100715.1
Francisella tularensis subsp. holarctica	Hare	Switzerland	B.49	SAMN08108643	PRJNA286987	GCA_002858385.1
Francisella tularensis subsp. holarctica	Hare	Switzerland	B.49	SAMN08108640	PRJNA286987	GCA_002858425.1
Francisella tularensis subsp. holarctica	Human	Switzerland	B.89	SAMN08108689	PRJNA286987	GCA_002857425.1
Francisella tularensis subsp. holarctica	Human	Switzerland	B.62	SAMN08108690	PRJNA286987	GCA_002857475.1
Francisella tularensis subsp. holarctica	Tick	Switzerland	B.61	SAMN08108673	PRJNA286987	GCA_002857765.1
Francisella tularensis subsp. holarctica	Tick	Switzerland	B.61	SAMN08108672	PRJNA286987	GCA_002857785.1
Francisella tularensis subsp. holarctica	Tick	Switzerland	B.61	SAMN08108671	PRJNA286987	GCA_002857805.1
Francisella tularensis subsp. holarctica	Human	Switzerland	B.45	SAMN08108648	PRJNA286987	GCA_002858265.1
Francisella tularensis subsp. holarctica	Hare	Switzerland	B.89	SAMN08108646	PRJNA286987	GCA_002858305.1
Francisella tularensis subsp. holarctica	Human	Switzerland	B.53	SAMN08108645	PRJNA286987	GCA_002858315.1
Francisella tularensis subsp. holarctica	Golden Lion Tamarin	Switzerland	B.46	SAMN08108642	PRJNA286987	GCA_002858345.1
Francisella tularensis subsp. holarctica	Marmoset	Switzerland	B.85	SAMN08108641	PRJNA286987	GCA_002858405.1
Francisella tularensis subsp. holarctica	Human	Switzerland	B.90	SAMN08108636	PRJNA286987	GCA_002858505.1
Francisella tularensis subsp. holarctica	European Hare	Spain	FDC423	SAMN03773895	PRJNA285145	GCA_002100845.1
Francisella tularensis subsp. holarctica	European Hare	Spain	F0783	SAMN03773734	PRJNA285145	GCA_002101185.1

Francisella tularensis subsp. holarctica	Hare	Switzerland	B.33	SAMN08108682	PRJNA286987	GCA_002857585.1
Francisella tularensis subsp. holarctica	Human	Switzerland	B.87	SAMN08108670	PRJNA286987	GCA_002857815.1
Francisella tularensis subsp. holarctica	Human	Switzerland	B.49	SAMN08108669	PRJNA286987	GCA_002857845.1
Francisella tularensis subsp. holarctica	Human	Switzerland	B.91	SAMN08108668	PRJNA286987	GCA_002857865.1
Francisella tularensis subsp. holarctica	Human	Switzerland	B.89	SAMN08108657	PRJNA286987	GCA_002858075.1
Francisella tularensis subsp. holarctica	European Hare	Germany	F0784	SAMN03773735	PRJNA285145	GCA_002101165.1
Francisella tularensis subsp. holarctica	Human	Spain	FDC434	SAMN03773906	PRJNA285145	GCA_002100625.1
Francisella tularensis subsp. holarctica	Human	Spain	FDC424	SAMN03773896	PRJNA285145	GCA_002100855.1
Francisella tularensis subsp. holarctica	Hare	Germany	08T0073	SAMN03773868	PRJNA353900	GCA_001953575.1
Francisella tularensis subsp. holarctica	Hare	Switzerland	B.90	SAMN08108680	PRJNA286987	GCA_002857625.1
Francisella tularensis subsp. holarctica	Hare	Switzerland	B.33	SAMN08108683	PRJNA286987	GCA_002857565.1
Francisella tularensis subsp. holarctica	Hare	Switzerland	B.45	SAMN08108684	PRJNA286987	GCA_002857545.1
Francisella tularensis subsp. holarctica	Monkey	Switzerland	B.33	SAMN08108687	PRJNA286987	GCA_002857465.1
Francisella tularensis subsp. holarctica	Marten	Switzerland	B.45	SAMN08108675	PRJNA286987	GCA_002857685.1
Francisella tularensis subsp. holarctica	Mouse	Switzerland	B.87	SAMN08108674	PRJNA286987	GCA_002857745.1
Francisella tularensis subsp. holarctica	Human	Switzerland	B.53	SAMN08108667	PRJNA286987	GCA_002857875.1
Francisella tularensis subsp. holarctica	Human	Switzerland	B.53	SAMN08108658	PRJNA286987	GCA_002858065.1
Francisella tularensis subsp. holarctica	Human	Switzerland	FDC310	SAMN03773858	PRJNA285145	GCA_002100405.1
Francisella tularensis subsp. holarctica	Arvicolinae sp.	Spain	FDC420	SAMN03773892	PRJNA285145	GCA_002100505.1
Francisella tularensis subsp. holarctica	European Hare	Spain	FDC413	SAMN03773886	PRJNA285145	GCA_002101015.1
Francisella tularensis subsp. holarctica	Human	Spain	FDC427	SAMN03773899	PRJNA285145	GCA_002100525.1
Francisella tularensis subsp. holarctica	Human	Spain	FDC431	SAMN03773903	PRJNA285145	GCA_002100775.1
Francisella tularensis subsp. holarctica	Edible dormouse	Germany	14T0103	SAMN09080665	PRJNA464279	GCA_003122155.1
Francisella tularensis subsp. holarctica	Human	Switzerland	B.85	SAMN08108647	PRJNA286987	GCA_002858285.1

Francisella tularensis subsp. holarctica	Marmoset	Germany	F0777	SAMN03773730	PRJNA285145	GCA_002100335.1
Francisella tularensis subsp. holarctica	Human	Switzerland	B.88	SAMN08108655	PRJNA286987	GCA_002858115.1
Francisella tularensis subsp. holarctica	Human	Switzerland	B.88	SAMN08108650	PRJNA286987	GCA_002858185.1
Francisella tularensis subsp. holarctica	Human	Switzerland	B.61	SAMN08108651	PRJNA286987	GCA_002858215.1
Francisella tularensis subsp. holarctica	Human	Netherlands	Francisella tularensis 15012907	SAMEA4765253	PRJEB27514	GCA_900491795.1
Francisella tularensis subsp. holarctica	Tick	Switzerland	B.62	SAMN08108664	PRJNA286987	GCA_002857945.1
Francisella tularensis subsp. holarctica	Human	Switzerland	B.46	SAMN08108652	PRJNA286987	GCA_002858205.1
Francisella tularensis subsp. holarctica	Human	Switzerland	B.33	SAMN08108656	PRJNA286987	GCA_002858105.1
Francisella tularensis subsp. holarctica	Human	Switzerland	B.33	SAMN08108654	PRJNA286987	GCA_002858165.1
Francisella tularensis subsp. holarctica	Human	Spain	FDC430	SAMN03773902	PRJNA285145	GCA_002100605.1
Francisella tularensis subsp. holarctica	European Hare	Spain	FDC415	SAMN03773887	PRJNA285145	GCA_002101005.1
Francisella tularensis subsp. holarctica	Raccoon Dog	Germany	FDC409	SAMN03773883	PRJNA285142	GCA_002102465.1
Francisella tularensis subsp. holarctica	Hare	Switzerland	B.45	SAMN08108688	PRJNA286987	GCA_002857445.1
Francisella tularensis subsp. holarctica	Hare	Switzerland	B.53	SAMN08108676	PRJNA286987	GCA_002857715.1
Francisella tularensis subsp. holarctica	UNK	Russia	SCPM-O-B-7554 (Kh-M 4)	SAMN10386538	PRJNA269675	GCA_003721555.1
Francisella tularensis subsp. holarctica	UNK	Russia	SCPM-O-B-7557 (Kh-M 8m)	SAMN10386540	PRJNA269675	GCA_003721615.1
Francisella tularensis subsp. holarctica	UNK	Russia	SCPM-O-B-7556 (Kh-M 8k)	SAMN10386539	PRJNA269675	GCA_003721625.1
Francisella tularensis subsp. holarctica	Tick	Switzerland	FT9C-G7	SAMN03774936	PRJNA286987	GCA_002027745.1
Francisella tularensis subsp. holarctica	Tick	Switzerland	FT8C-4F	SAMN03774935	PRJNA286987	GCA_002027765.1
Francisella tularensis subsp. holarctica	Tick	Switzerland	B.59	SAMN08108665	PRJNA286987	GCA_002857915.1
Francisella tularensis subsp. holarctica	Tick	Switzerland	B.59	SAMN08108663	PRJNA286987	GCA_002857955.1
Francisella tularensis subsp. holarctica	Human	Switzerland	B.87	SAMN08108649	PRJNA286987	GCA_002858245.1
Francisella tularensis subsp. holarctica	Tick	France	11-789-5S	SAMEA3593398	PRJEB11276	GCA_900016295.2
Francisella tularensis subsp. holarctica	Tick	Switzerland	B.45	SAMN08108662	PRJNA286987	GCA_002858015.1

Francisella tularensis subsp. holarctica	Human	Switzerland	B.88	SAMN08108637	PRJNA286987	GCA_002858465.1
Francisella tularensis subsp. holarctica	Tick	Switzerland	B.62	SAMN08108666	PRJNA286987	GCA_002857905.1
Francisella tularensis subsp. holarctica	Tick	Switzerland	B.62	SAMN08108661	PRJNA286987	GCA_002857985.1
Francisella tularensis subsp. holarctica	Tick	Switzerland	B.62	SAMN08108660	PRJNA286987	GCA_002858005.1
Francisella tularensis subsp. holarctica	Tick	Switzerland	B.62	SAMN08108659	PRJNA286987	GCA_002858045.1
Francisella tularensis subsp. holarctica	European Hare	Spain	FDC418	SAMN03773890	PRJNA285145	GCA_002100485.1
Francisella tularensis subsp. holarctica	Human	Spain	FDC433	SAMN03773905	PRJNA285145	GCA_002100785.1
Francisella tularensis subsp. holarctica	Hare	Switzerland	FDC306	SAMN03773857	PRJNA285145	GCA_002101045.1
Francisella tularensis subsp. holarctica	Human	Switzerland	FT-32	SAMN03774932	PRJNA286987	GCA_002027725.1
Francisella tularensis subsp. holarctica	Human	Switzerland	B.46	SAMN08108653	PRJNA286987	GCA_002858145.1
Francisella tularensis subsp. holarctica	Hare	France	F0295	SAMN03773721	PRJNA285145	GCA_002100175.1
Francisella tularensis subsp. holarctica	Fox	Germany	FDC407	SAMN03773881	PRJNA285142	GCA_002102515.1
Francisella tularensis subsp. holarctica	UNK	Russia	SCPM-O-B-7562 (Kh-M 26/2)	SAMN10386541	PRJNA269675	GCA_003721535.1
Francisella tularensis subsp. holarctica	Human	Netherlands	Francisella tularensis 16007282	SAMEA4765254	PRJEB27514	GCA_900491815.1
Francisella tularensis subsp. holarctica	Brown Hare	France	11-935-13S	SAMEA3593396	PRJEB11276	GCA_900015305.2
Francisella tularensis subsp. holarctica	Brown Hare	France	11-930-9S	SAMEA3593397	PRJEB11276	GCA_900016285.2
Francisella tularensis subsp. holarctica	European Hare	Germany	F0781	SAMN03773732	PRJNA285145	GCA_002100185.1
Francisella tularensis subsp. holarctica	Macaque	Germany	F0775	SAMN03773729	PRJNA285145	GCA_002100375.1
Francisella tularensis subsp. holarctica	European Hare	Spain	FDC429	SAMN03773901	PRJNA285145	GCA_002100795.1
Francisella tularensis subsp. holarctica	Castor sp	Germany	FDC408	SAMN03773882	PRJNA285142	GCA_002102455.1
Francisella tularensis subsp. holarctica	Human	Netherlands	Francisella tularensis X17000610	SAMEA4765256	PRJEB27514	GCA_900491805.1
Francisella tularensis subsp. holarctica	Human	Spain	FDC432	SAMN03773904	PRJNA285145	GCA_002100565.1
Francisella tularensis subsp. holarctica	Human	Spain	FDC426	SAMN03773898	PRJNA285145	GCA_002100765.1
Francisella tularensis subsp. holarctica	European Hare	Spain	FDC416	SAMN03773888	PRJNA285145	GCA_002100955.1

Francisella tularensis subsp. holarctica	European Hare	Spain	FDC417	SAMN03773889	PRJNA285145	GCA_002100935.1
Francisella tularensis subsp. holarctica	Human	Switzerland	FDC305	SAMN03773856	PRJNA285145	GCA_002101085.1
Francisella tularensis subsp. holarctica	Human	Switzerland	B.46	SAMN08108644	PRJNA286987	GCA_002858365.1
Francisella tularensis subsp. holarctica	Hare	Germany	FDC338	SAMN03773859	PRJNA285145	GCA_002100415.1
Francisella tularensis subsp. holarctica	Arvicolinae	Spain	FDC428	SAMN03773900	PRJNA285145	GCA_002100535.1
Francisella tularensis subsp. holarctica	European Hare	Spain	FDC412	SAMN03773885	PRJNA285145	GCA_002100455.1
Francisella tularensis subsp. holarctica	Hare	Switzerland	B.85	SAMN08108639	PRJNA286987	GCA_002858485.1
Francisella tularensis subsp. holarctica	European Hare	Spain	FDC419	SAMN03773891	PRJNA285145	GCA_002100945.1
Francisella tularensis subsp. holarctica	Natural spring water	Italy	F0732	SAMN03773728	PRJNA285145	GCA_002100365.1
Francisella tularensis subsp. holarctica	Tick	Russia	SCPM-O-B-7178(A-1045)	SAMN10924161	PRJNA269675	GCA_004214255.1
Francisella tularensis subsp. holarctica	Hare	Germany	FDC300	SAMN03773854	PRJNA285145	GCA_002101105.1
Francisella tularensis subsp. holarctica	European Hare	Spain	FDC421	SAMN03773893	PRJNA285145	GCA_002100925.1
Francisella tularensis subsp. holarctica	European Hare	Germany	F0624	SAMN03773725	PRJNA285145	GCA_002100195.1
Francisella tularensis subsp. holarctica	Human	Spain	FDC425	SAMN03773897	PRJNA285145	GCA_002100865.1
Francisella tularensis subsp. holarctica	Human	Spain	FDC422	SAMN03773894	PRJNA285145	GCA_002100875.1
Francisella tularensis subsp. holarctica	Human	Switzerland	B.85	SAMN08108638	PRJNA286987	GCA_002858435.1
Francisella tularensis subsp. holarctica	Human	France	FDC113	SAMN03773839	PRJNA285145	GCA_002100445.1
Francisella tularensis subsp. holarctica	European Hare	Germany	F0608	SAMN03773724	PRJNA285145	GCA_002100165.1
Francisella tularensis subsp. holarctica	European Hare	Spain	FDC411	SAMN03773884	PRJNA285145	GCA_002101035.1
Francisella tularensis subsp. holarctica	Human	Switzerland	FDC304	SAMN03773855	PRJNA285145	GCA_002101095.1
Francisella tularensis subsp. holarctica	Human	France	FDC095	SAMN03773837	PRJNA285145	GCA_002100325.1
Francisella tularensis subsp. holarctica	Human	France	FDC099	SAMN03773838	PRJNA285145	GCA_002101125.1
Francisella tularensis subsp. holarctica	Water	Sweden	R13-38	SAMN03249218	PRJNA269095	GCA_000807905.1
Francisella tularensis subsp. holarctica	Hare	Spain	FSC455	SAMN03774110	PRJNA285145	GCA_002100645.1

Francisella tularensis subsp. holarctica 257	UNK	USA	257	SAMN02595214	PRJNA18251	GCA_000153845.1
Francisella tularensis subsp. holarctica BD11-00177	Human	Netherlands	BD11-00177	SAMN02690826	PRJNA177784	GCA_000681495.1
Francisella tularensis subsp. holarctica F92	Marmoset	Germany	F92	SAMN02603045	PRJNA175244	GCA_000313385.1
Francisella tularensis subsp. holarctica FSC022		USA	FSC022	SAMN02595215	PRJNA19015	GCA_000154145.1
Francisella tularensis subsp. holarctica FSC022	Human	Japan	FSC022	SAMN06075683	PRJNA355394	GCA_002027465.1
Francisella tularensis subsp. holarctica FSC200	Human	Sweden	FSC200	SAMN01085703	PRJNA16087	GCA_000168775.2
Francisella tularensis subsp. holarctica FTNF002-00	Human	France	FTNF002-00	SAMN02603037	PRJNA20197	GCA_000017785.1
Francisella tularensis subsp. holarctica LVS	UNK		LVS	SAMEA3138197	PRJNA16421	GCA_000009245.1
Francisella tularensis subsp. holarctica LVS	UNK		LVS	SAMN03010443	PRJNA236485	GCA_000833335.1
Francisella tularensis subsp. holarctica OSU18	UNK	OK	OSU18	SAMN02641482	PRJNA32025	GCA_000011405.1
Francisella tularensis subsp. holarctica OSU18	UNK	OK	OSU18	SAMN02641482	PRJNA17265	GCA_000014605.1
Francisella tularensis subsp. holarctica PHIT-FT049	UNK	Turkey	PHIT-FT049	SAMN02641556	PRJNA230014	GCA_000524575.1
Francisella tularensis subsp. holarctica URFT1	Human	Sweden	URFT1	SAMN02471335	PRJNA19645	GCA_000170295.1
Francisella tularensis subsp. mediasiatica	Tick	Russia	SCPM-O-B-7176(A-678)	SAMN10924159	PRJNA269675	GCA_004213485.1
Francisella tularensis subsp. mediasiatica	Tick	Russia	SCPM-O-B-7175(A-554)	SAMN10924158	PRJNA269675	GCA_004296555.1
Francisella tularensis subsp. mediasiatica	Siberian red vole	Russia	SCPM-O-B-7177(A-823)	SAMN10924160	PRJNA269675	GCA_004214305.1
Francisella tularensis subsp. mediasiatica FSC147	Midday gerbil	Europe	FSC147; GIEM 543	SAMN02604345	PRJNA19571	GCA_000018925.1
Francisella tularensis subsp. novicida	Human	TX	TCH2015	SAMN06481648	PRJNA378245	GCA_002952075.1
Francisella tularensis subsp. novicida	Human	AL	AL97-2214	SAMN03107513	PRJNA260088	GCA_001880205.1
Francisella tularensis subsp. novicida	Human	AZ	AZ06-7470	SAMN03107514	PRJNA260086	GCA_001880245.1
Francisella tularensis subsp. novicida		UT	FAI	SAMN02870033	PRJNA244559	GCA_000742085.1
Francisella tularensis subsp. novicida	Water	UT	DPG 3A-IS	SAMN02769653	PRJNA240119	GCA_000834965.1
Francisella tularensis subsp. novicida D9876	Human	LA	D9876	SAMN03092276	PRJNA235892	GCA_000833355.1
Francisella tularensis subsp. novicida F6168	Human	CA	F6168	SAMN03010444	PRJNA236045	GCA_000833165.1
Francisella tularensis subsp. novicida FTE	UNK	USA	FTE	SAMN02469397	PRJNA30119	GCA_000155755.1
Francisella tularensis subsp. novicida FTG	Hotsprings	UT	FTG	SAMN02469396	PRJNA30447	GCA_000156415.1

Francisella tularensis subsp. novicida GA99-3548	UNK	USA	GA99-3548	SAMN02595218	PRJNA19573	GCA_000154265.1
Francisella tularensis subsp. novicida GA99-3549	UNK	USA	GA99-3549	SAMN02595217	PRJNA19019	GCA_000154185.1
Francisella tularensis subsp. novicida PA10-7858	Human	PA	PA10-7858	SAMN05449121	PRJNA215224	GCA_001865695.1
Francisella tularensis subsp. novicida U112	Saltwater	UT	U112	SAMN03107387	PRJNA236529	GCA_000833375.1
Francisella tularensis subsp. novicida U112	UNK	USA	U112	SAMN02604254	PRJNA16088	GCA_000014645.1
Francisella tularensis subsp. tularensis	Human	WY	WY96	SAMN03817044	PRJNA288604	GCA_001262115.1
Francisella tularensis subsp. tularensis	Human	OH	NIH B-38	SAMN03219196	PRJNA240113	GCA_000833475.1
Francisella tularensis subsp. tularensis	Human	OH	Scherm	SAMN02769658	PRJNA240118	GCA_000833555.1
Francisella tularensis subsp. tularensis 1378	Human	NM	1378	SAMN02469750	PRJNA189042	GCA_000380385.1
Francisella tularensis subsp. tularensis 3571	Human	NM	3571	SAMN02469753	PRJNA189041	GCA_000346525.1
Francisella tularensis subsp. tularensis 70001275	Human	UT	70001275	SAMN02469747	PRJNA170180	GCA_000313275.1
Francisella tularensis subsp. tularensis 70102010	Human	UT	70102010	SAMN02469756	PRJNA170747	GCA_000305835.1
Francisella tularensis subsp. tularensis 79201237	Human	UT	79201237	SAMN02469752	PRJNA189048	GCA_000380425.1
Francisella tularensis subsp. tularensis 80700069	Human	UT	80700069	SAMN02469751	PRJNA189049	GCA_000380445.1
Francisella tularensis subsp. tularensis 80700075	Human	UT	80700075	SAMN02469749	PRJNA189044	GCA_000380405.1
Francisella tularensis subsp. tularensis 80700075	Human	UT	80700075	SAMN02469757	PRJNA170750	GCA_000305875.1
Francisella tularensis subsp. tularensis 80700103	Human	UT	80700103	SAMN02469755	PRJNA170749	GCA_000305855.1
Francisella tularensis subsp. tularensis 831	Human	NM	831	SAMN02469748	PRJNA170751	GCA_000305895.1
Francisella tularensis subsp. tularensis AS_713	Rabbit	NM	AS_713	SAMN02469754	PRJNA170752	GCA_000305915.1
Francisella tularensis subsp. tularensis FSC033	UNK	USA	FSC033	SAMN02595216	PRJNA19017	GCA_000154165.1
Francisella tularensis subsp. tularensis FSC198	UNK	Slovakia	FSC 198	SAMEA3138200	PRJNA17375	GCA_000009325.1
Francisella tularensis subsp. tularensis MA00-2987	UNK	USA	MA00-2987	SAMN02595231	PRJNA261847	GCA_001267475.1
Francisella tularensis subsp. tularensis MA00-2987	UNK	USA	MA00-2987	SAMN02595231	PRJNA30443	GCA_000155535.1
Francisella tularensis subsp. tularensis NE061598	UNK	NE	NE061598	SAMN02604230	PRJNA38289	GCA_000023305.1

Francisella tularensis subsp. tularensis SCHU S4	UNK	Europe	SCHU S4	SAMEA3138185	PRJNA9	GCA_000008985.1
Francisella tularensis subsp. tularensis SCHU S4	Human	OH	SHU-S4	SAMN03246840	PRJNA239340	GCA_000833535.1
Francisella tularensis subsp. tularensis str. SCHU S4 substr. FSC237	UNK	USA	Schu S4 substr. FSC043	SAMN02335350	PRJNA217352	GCA_000628945.1
Francisella tularensis subsp. tularensis str. SCHU S4 substr. FTS-634/635	UNK	USA	Schu S4 substr. FTS-634/635	SAMN02335351	PRJNA217353	GCA_000628905.1
Francisella tularensis subsp. tularensis str. SCHU S4 substr. NR-10492	UNK	USA	Schu S4 substr. NR-10492	SAMN02335347	PRJNA217349	GCA_000629005.1
Francisella tularensis subsp. tularensis str. SCHU S4 substr. NR-28534	UNK	USA	SCHU S4 substr. NR-28534	SAMN03264783	PRJNA270247	GCA_000978785.2
Francisella tularensis subsp. tularensis str. SCHU S4 substr. NR-28534	UNK	USA	Schu S4 substr. NR-28534	SAMN02335346	PRJNA217348	GCA_000628925.1
Francisella tularensis subsp. tularensis str. SCHU S4 substr. NR-643	UNK	USA	Schu S4 substr. NR-643	SAMN02335348	PRJNA217350	GCA_000628985.1
Francisella tularensis subsp. tularensis str. SCHU S4 substr. SL	UNK	USA	Schu S4 substr. SL	SAMN02335349	PRJNA217351	GCA_000628965.1
Francisella tularensis subsp. tularensis TI0902	UNK	USA	TI0902	SAMN02604300	PRJNA64439	GCA_000248435.2
Francisella tularensis subsp. tularensis TIGB03	UNK	USA	TIGB03	SAMN02604301	PRJNA64441	GCA_000248415.2
Francisella tularensis subsp. tularensis WY-00W4114	Prairie Dog	WY	WY-00W4114	SAMN03266146	PRJNA81045	GCA_001011135.1
Francisella tularensis subsp. tularensis WY96-3418	UNK	USA	WY96-3418	SAMN02603036	PRJNA18459	GCA_000016105.1

Supplementary table 2: ORFs and Virulence factors annotated from draft genomes and compared with F. *tularensis subsp. tularensis* SCHU S4

VF class	Virulence factors	Related genes	OADDL-FT1-Narayanan	OADDL-FT2-Couger	OADDL-FT3-Bates	F.tularensis subsp. tularensis SCHU S4
Adherence and invasion	EF-Tu	tufA	orf01865	orf01834	ORF01415	FTT_0137
	FsaP	fsaP	orf01887	orf01857	ORF01440	FTT_0119
	Type IV pili	Undetermined	orf00990	orf00962	ORF00752	FTT_0905
		pilB	orf00723	orf00692	ORF01016	FTT_1133
		pilC	orf00722	orf00691	ORF01017	FTT_1134
		pilD	orf01254	orf01219	ORF00498	FTT_0683c
		pilE1/pilA	orf01005	orf00978	ORF00737	FTT_0890c
		pilE2/pilE	orf01006	orf00979	ORF00736	FTT_0889c
		pilE3/pilV	orf01007	orf00980	ORF00735	FTT_0888c
		pilE4	orf01039	orf01012	ORF00703	FTT_0861c
		pilE5	orf01759	orf01726	ORF01304	FTT_0230c
		pilE6	orf00514	orf00479	ORF00059	FTT_1314c
		pilF	orf00815	orf00787	ORF00923	FTT_1057c
		pilN	orf00695	orf00658	ORF01049	FTT_1159c
		pilO	orf00696	orf00659	ORF01048	FTT_1158c
		pilP	orf00697	orf00660	ORF01047	FTT_1157c
		pilQ	orf00698	orf00661	ORF01046	FTT_1156c
		pilT	orf01924	orf01894	ORF01478	FTT_0088
Intracellular survival	Acid phosphotase	acpA	orf01769	orf01736	orf01314	FTT_0221
		acpB	orf01846	orf01814	orf01396	FTT_0156
		acpC	orf01323	orf01289	orf00430	FTT_0620
		hapA	orf00806	orf00778	orf00932	FTT_1064*
	DipA	dipA	orf01613	orf01579	orf00134	FTT_0369c
	OmpA	ompA	orf01074	orf01044	orf00669	FTT_0831c
	RipA	ripA	orf01816	orf01784	orf01364	FTT_0181c
Iron uptake	Ferrous iron-transport system	feoB	orf01741	orf01708	orf01285	FTT_0249
		fupA	orf00977	orf00949	orf00765	FTT_0918
	Francisella siderophore locus	fslA	orf01987	orf01960	orf01546	FTT_0029c
		fslB	orf01988	orf01961	orf01547	FTT_0028c
		fslC	orf01989	orf01962	orf01548	FTT_0027c
		fslD	orf01990	orf01963	orf01549	FTT_0026c

		fslE	orf01991	orf01964	orf01550	FTT_0025c
Magnesium uptake	FmvB	fmvB	orf01342	orf01308	orf00410	FTT_0602c
Nutritional virulence	Arginine transporter	argP	orf00917	orf00890	orf00823, orf00824	FTT_0968c
	Asparagine transporter AnsP	ansP	orf01874	orf01844	orf01425, orf01426	FTT_0129
	Biotin metabolism	bioA	orf00954	orf00927	orf00787	FTT_0938
		bioB	orf00955	orf00928	orf00786	FTT_0937c
		bioC	orf00957	orf00930	orf00784	FTT_0935c
		bioD	orf00958	orf00931	orf00783	FTT_0934c
		bioF	orf00956	orf00929	orf00785	FTT_0936c
		bioJ	orf00951	orf00924	orf00790	FTT_0941c
		birA	-	-	-	-
		bplA	orf01495	orf01461	orf00255	FTT_0477c
	Cysteine acquisition	ggt	orf00665	orf00629	orf01077	FTT_1181c
	Glutamate transporter GadC	gadC	orf01492	orf01458	orf00258	FTT_0480c
	Isoleucine transporter	ileP	orf01957	orf01929	orf01512	FTT_0056c
	Purine	purCD	orf01001	orf00973	orf00741	FTT_0894
		purM	orf01002	orf00974	orf00740	FTT_0893
	Pyrimidine biosynthesis	carA	orf00072	orf00354	orf01785	FTT_1663
		carB	orf00071	orf00355	orf01784	FTT_1664
		pyrB	orf00070	orf00356	orf01783	FTT_1665
Secretion system	T6SS (FPI)	dotU/tssL	orf00018; orf00447; orf00476; orf02134	orf00406; orf00440	ORF00018, orf01697, orf01726, orf02159	FTT_1351; FTT_1706
		iglA/tssB	orf00007; orf00436; orf00466; orf02126	orf00414; orf00430	ORF00008, ORF01687, ORF01714, ORF02150	FTT_1359c; FTT_1714c
		iglB/tssC	orf00008; orf00437; orf00467; orf02127	orf00413; orf00431	ORF00009,ORF01688, ORF01715, ORF02151	FTT_1358c; FTT_1713c
		iglC/hcp	orf00009; orf00438; orf00468; orf02128	orf00412; orf00432	ORF00010, ORF01689, ORF01715, ORF02151	FTT_1357c; FTT_1712c
		iglD/tssK	orf00010; orf00439;	orf00411; orf00433	ORF00011, ORF01690,	FTT_1356c; FTT_1711c

			orf00469;<br>orf02129		ORF01717,<br>ORF02153	
		iglE/tssJ	orf00023;<br>orf00453;<br>orf00481;<br>orf02139	orf00401;<br>orf00446	ORF1703,<br>ORF01733,<br>ORF02166	FTT_1346;<br>FTT_1701
		iglF/clpV	orf00021;<br>orf00479;<br>orf02137	orf00403;<br>orf00443	ORF00022,<br>ORF00023,<br>ORF01700,<br>ORF01730,<br>ORF02163,<br>ORF022164	FTT_1348;<br>FTT_1703
		iglG	orf00020;<br>orf00449;<br>orf00478;<br>orf02136	orf00404;<br>orf00442	ORF00021,<br>ORF01699,<br>ORF01729,<br>ORF02162	FTT_1349;<br>FTT_1704
		iglH	orf00019;<br>orf00448;<br>orf00477;<br>orf02135	orf00405;<br>orf00441	ORF00019,<br>ORF00020,<br>ORF01698,<br>ORF01728,<br>ORF02160	FTT_1350;<br>FTT_1705
		iglI	orf00017;<br>orf00446;<br>orf00475;<br>orf02133	orf00407;<br>orf00439	ORF00017,<br>ORF01696,<br>ORF01725,<br>ORF02158	FTT_1352;<br>FTT_1707
		iglJ	orf00016;<br>orf00445;<br>orf00474;<br>orf02132	orf00408;<br>orf00438	ORF00016,<br>ORF01695,<br>ORF02157	FTT_1353;<br>FTT_1708
		pdpA	orf00025;<br>orf00455;<br>orf00483;<br>orf02141	orf00398;<br>orf00448	ORF00027,<br>ORF01705,<br>ORF01736	FTT_1344;<br>FTT_1699
		pdpB	orf00024;<br>orf00454;<br>orf00482;<br>orf02140	orf00447	ORF00026,<br>ORF01704,<br>ORF01734,<br>ORF01735,<br>ORF02168	FTT_1345;<br>FTT_1700
		pdpC	orf00471;<br>orf02131	orf00409;<br>orf00436	ORF00014,<br>ORF01694,<br>ORF02156	FTT_1354;<br>FTT_1709
		pdpD	orf00006;<br>orf00435;<br>orf00465;<br>orf02125	orf00415;<br>orf00429	ORF00007,<br>ORF01694,<br>ORF02156	FTT_1360c;<br>FTT_1715c
		pdpE	orf00011;<br>orf00440;<br>orf00470	orf00410;<br>orf00434	ORF00012,<br>ORF01692,<br>ORF01718,<br>ORF02154	FTT_1355;<br>FTT_1710

		vgrG	orf00022; orf00452; orf00480; orf02138	orf00402; orf00445	ORF00024, ORF01702, ORF01732, ORF02165	FTT_1347; FTT_1702
Serum resistance and immune evastion	Capsule	Undetermined	orf01125	orf01093	orf00624	FTT_0790
		Undetermined	orf01122	orf01091	orf00626	FTT_0792
		Undetermined	orf01121	orf01090	orf00627	FTT_0793
		Undetermined	orf01118	orf01088	orf00628	FTT_0794
		Undetermined	orf01117	orf01087	orf00629	FTT_0795
		Undetermined	orf01116	orf01086	orf00630	FTT_0796
		Undetermined	orf01115	orf01085	orf00631	FTT_0797
		Undetermined	orf01114	orf01084	orf00632	FTT_0798
		Undetermined	orf01113	orf01083	orf00633	FTT_0799
		Undetermined	orf01112	orf01082	orf00634	FTT_0800
		capA	orf01105	orf01075	orf00641	FTT_0807
		capB	orf01107	orf01077	orf00639	FTT_0805
		capC	orf01106	orf01076	orf00640	FTT_0806
		galE	orf01123	orf01092	orf00625	FTT_0791
		rpe	orf01126	orf01094	orf00623	FTT_0789
		waaL/rfaL	orf00599	orf00564	orf01145	FTT_1238c
		waaY	orf00601	orf00566	orf01143	FTT_1236
	LPS	flmF2	orf01520	orf01484	orf00231	FTT_0454
		flmK	orf01519	orf01483	orf00232	FTT_0455c
		kdtA	orf00195	orf00233	orf01910	FTT_1561
		lpcC	orf00602	orf00567	orf01142	FTT_1235c
		lpxA/glmU	orf01592	orf01557	orf00155	FTT_0387
		lpxD1	orf00185	orf00243	orf01900	FTT_1571c
		lpxD2	orf01702	orf01668	orf01245	FTT_0286c
		lpxE	orf01004	orf00977	orf00738	FTT_0891
		lpxF	-	-	-	-
		manB	orf00330	orf00100	orf02045	FTT_1447c
		manC	orf00329	orf00101	orf02044	FTT_1448c
		waaZ	orf00600	orf00565	orf01144	FTT_1237
		wbtA	orf00309	orf00120	orf02022	FTT_1464c
		wbtB	orf00310	orf00119	orf02023	FTT_1463c

		wbtC	orf00311	orf00118	orf02024	FTT_1462c
		wbtD	orf00312	orf00117	orf02026	FTT_1461c
		wbtE	orf00313	orf00116	orf02027	FTT_1460c
		wbtF	orf00314	orf00115	orf02028	FTT_1459c
		wbtG	orf00318	orf00113	orf02031	FTT_1457c
		wbtH	orf00319	orf00112	orf02032	FTT_1456c
		wbtI	orf00320	orf00111	orf02033	FTT_1455c
		wbtJ	orf00321	orf00109	orf02034	FTT_1454c
		wbtK	orf00323	orf00107	orf02037	FTT_1452c
		wbtL	orf00324	orf00106	orf02038	FTT_1451c
		wbtM	orf00326	orf00104	orf02041	FTT_1450c
		wbtN	-	-	-	-
		wbtO	-	-	-	-
		wbtP	-	-	-	-
		wbtQ	-	-	-	-
		wzx	orf00322	orf00108	orf02035	FTT_1453c
		wzy	orf00315	orf00114	orf02029	FTT_1458c
Toxin	Phytotoxin phaseolotoxin(Pseudomonas)	cysC1	orf00825	orf00798	orf00914	-
